# Focused Subspecialty Training in Plastic Surgery Residency: An Objective Assessment of the Cleveland Clinic Pilot Program

**DOI:** 10.1093/asjof/ojaf040

**Published:** 2025-05-12

**Authors:** Demetrius M Coombs, Steven L Bernard, Raymond Isakov, James E Zins

## Abstract

**Background:**

Many senior-level plastic surgery residents have a firm idea regarding the complexion of their future practice.

**Objectives:**

Rather than continuing with traditional rotations in the sixth year, it is suggested that chief residents have the option of a 6-month focused training experience in their final year of plastic surgery training.

**Methods:**

A pilot program of focused training in the subspecialty area of the chief resident's interest was initiated at our institution in July 2022. Areas of focus began with aesthetic surgery, whereas hand/microsurgery, gender-affirming, and craniofacial surgery were under development. Objective means of assessing the value of the resident's experience were developed, including blinded program surveys completed by participating and nonparticipating residents and attending surgeons, case log activity, and academic productivity.

**Results:**

Objective assessment demonstrated that the pilot was favorably received by all residents and participating staff. All residents expressed interest in participating during their chief year and requested the program continue. Furthermore, 10 (48%) residents were interested in pursuing a formal fellowship different than their focused training experience. Attendees unanimously agreed that the pilot offered a substantial advantage and improvement to the current residency training model and supported its continuation. Notably, there were no major criticisms, logistical problems, or administrative burdens encountered.

**Conclusions:**

Given the increasing complexity of plastic surgery practice and rising competition from other specialties, a focused training experience before postgraduate fellowship or formal practice may represent an alternative means of enhancing plastic surgery education. The current pilot attempts to test and objectify this concept.

**Level of Evidence: 5 (Therapeutic):**

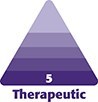

Plastic surgery resident education requires exposure to an increasing array of complex problems in a broad range of surgical disciplines. At the same time, these disparate fields overlap with our sister subspecialties, some of which are more limited in scope and more anatomically focused. This diverse nature of our specialty makes mastering all areas of plastic surgery a significant challenge. Few of us are able to master them all.

Integrated residents are a sophisticated group. Most senior residents have a clear idea of the focus of their future practice, and many graduates choose to limit or focus their practice on specific areas of plastic surgery rather than attempt to cover it in its entirety. If that is the case, perhaps repeating in the PGY6 year much of what has been done in PGY4-PGY5 may not be the most effective use of resident time and education. There have been recent efforts that attempt to streamline neurosurgical and plastic surgery training, including the notion of competency-based education (CBE).^1,2^ We suggest an alternative approach: an in-depth, focused opportunity in 1 area of plastic surgery of the resident's choosing might provide a more satisfying and effective experience and may better prepare the PGY6 resident for their future independent practice. We suggest a 6-month pilot consisting of an in-depth, focused training in a single area of plastic surgery during the PGY6 year. Our plan is designed as an educational commitment only. No formal recognition or certificate would be provided. Subspecialty options would be specific to a given residency depending on the subspecialty clinical strengths of that particular program.

In the text that follows, we describe our institutional experience with the notion of focused subspecialty training in plastic surgery using our aesthetic module as a template. We also provide an objective outcome assessment to suggest additions for the future. Our goal is to offer a proposed framework that may aid other programs wishing to establish a similar initiative.

## METHODS

This concept was first proposed at the 2021 American Board of Plastic Surgery (ABPS) Directors' Meeting, where an ad hoc aesthetic committee was established to address diplomate concerns and issues, one of which was education. Although the concept was first developed as focused training in aesthetic surgery, it became apparent that a more appropriate approach would be to expand the concept to focused training in plastic surgery. This would include other subspecialty modules and interests, including, but not necessarily limited to hand, craniofacial, and microsurgery. Additionally, this would give an opportunity for focused training to be available to a broader resident audience. Our aesthetic module was formulated in detail and initiated in July 2022. Modules in hand, craniofacial, and microsurgery are currently in the process of development.

A pilot program of focused training in the area of resident interest was approved by our institution's resident review committee. The pilot was also approved by the ABPS. Additional subspecialty modules were in stages of development, and the program was begun with the aesthetic module in this first pilot year. The duration of the experience was 6 months. Participants were required to complete rotations within the enterprise health system. Rotations outside our health system were not permitted. Night and weekend call responsibilities were unchanged in order to minimize disruptions and maintain service obligations.

Participation in the pilot was voluntary. Pilot requirements included completion of Plastic Surgery Operative Log minimums by the end of the PGY5 year, a mean annual In-Service Exam (ISE) score above the 50th percentile, successful progression through Accreditation Council for Graduate Medical Education milestones, and residency Program Director (PD) approval.

The structure for the focused training module in aesthetics followed a daily clinical block diagram and included 4 days of subspecialty training and 1 day of research per week, 1 participating resident-led advanced didactic lecture during the 6-month period, and clinical research responsibilities, including active participation in bi-weekly research meetings. The experience was designed as an apprenticeship model, with 2 month blocks, and included multiple staff. The resident was further required to submit a minimum of 1 clinical project to the annual American Society of Plastic Surgeons, American Association of Plastic Surgeons, or an alternative but relevant meeting. Finally, the operative log over the 6-month pilot was to be submitted to the residency program coordinator.

Given that the majority of facial aesthetic procedures are bilateral in nature, the senior surgeon typically performed the first side, and the focused training resident completed the second under close supervision. This was also the protocol for less invasive procedures, such as chemical peels, laser resurfacing, and facial fat injections; however, other injectables were predominantly performed by the attending surgeon in the office. Cases were reviewed before surgery with the senior surgeon, including the physical examination findings and preoperative photographs. One key benefit of the focused training experience is the opportunity to see the patient before surgery by participating in the examination, follow the postoperative course (including a re-examination of the before and after photographs), and help manage any postoperative complications that might arise. Although the senior residents had their own cosmetic clinic as part of the chief year, this was separate and divorced from the focused training experience.

A detailed proposal for focused training was developed, including rationale, description of the program, structure, goals and objectives, anticipated outcomes, program assessment, and measured learner outcomes. Faculty development to ensure faculty members understand goals and objectives and methods for effective program assessment was also detailed (Appendix A).

Objective means of assessing the value of the resident's experience were developed. This included a blinded program survey completed by the participating resident, nonparticipating PGY2-PGY6 residents, participating attending surgeons, and the PD and associate PD (Appendices B, C). Surveys were formatted in a manner comparable with the annual training program surveys distributed electronically at our institution with only slight modifications (Microsoft Forms, Redmond, WA) and completed over the course of 1 week in March 2023. Additional objective assessment included an examination of case log activity and academic productivity.

## RESULTS

### Nonparticipating Resident Survey

**Figure 1.**
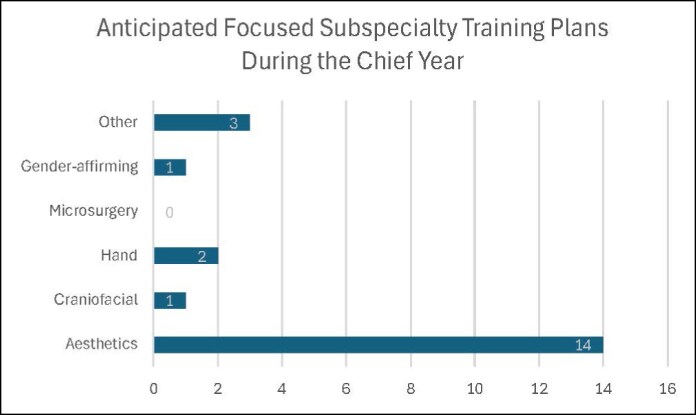
Areas of interest and anticipated plans among PGY2-6 residents for the focused training experience during their chief year. All residents surveyed (100%) were interested in the opportunity.

**Figure 2.**
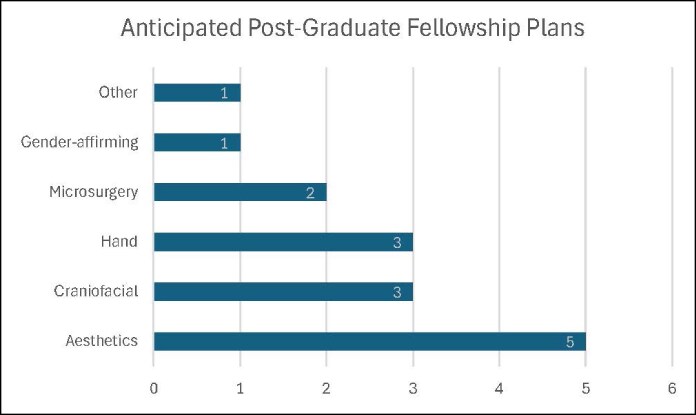
Anticipated fellowship plans after graduation. Fifteen residents (71%) were interested in pursuing a formal fellowship following their residency, demonstrating a broad range of interests.

Twenty-one PGY2-6 residents responded (100% response rate). All residents (100%) were interested in the focused subspecialty training experience during their sixth year. A breakdown of areas of interest for focused training is detailed in Figure 1. Fifteen (71%) were interested in pursuing a formal fellowship postresidency (Figure 2).

Ten residents (48%) were interested in pursuing a formal postresidency fellowship *different* than their anticipated focused training experience. Twenty (95%) indicated that the focused training pilot either never or rarely created any additional work or stress over the course of its duration. All (100%) recommended the program continue. Comments from the nonparticipating residents are provided in Table 1.

**Table 1.** Comments From Nonparticipating Residents and Participating Staff

Nonparticipating residents
Augments experience in a second field of plastic surgery (particularly if planning to complete a formal postgraduate fellowship) Allows for a broad focus early in residency and a targeted focus before graduation Excellent medical student recruitment tool Could represent the future of plastic surgery residency training Better develop curricula in other fields of plastic surgery (hand, microsurgery, craniomaxillofacial, and gender-affirming surgery) Optimize scheduling and rotations Refine requirements for eligibility to participate
Participating staff
Sophisticated and nuanced approach to an area of interest Helpful for parties to learn from one another Good idea that will improve with maturation Beneficial and should maintain commitment to other services as well Allows for enhanced focus and academic pursuits May require enhanced coordination in larger programs Establish a formal approval process for those who wish to be enrolled Consider 3-month rotations Expand to include more staff Develop additional subspecialty areas

### Participating Staff Surveys

Six staff responded (100% response rate). All staff (100%) agreed that the focused training pilot enhanced the resident's experience. All (100%) agreed that the pilot offers a significant advantage and improvement to the current residency training paradigm at our institution. All (100%) agreed that the program continue. Comments from participating staff also appear in Table 1.

### Participating Resident Evaluations

On the postfocused program survey, the participating resident indicated that the clinical volume and diversity of cases exceeded expectations, opportunities for patient evaluation and postoperative follow-up were educational, and the experience increased their fund of knowledge, technical expertise, and nuances and complexities of patients and surgical cases. It was also reported that the presence of fellows and additional residents in cases and clinics was not felt to have had a detrimental impact in terms of the focused training experience. In summary, the resident found the program to be extremely valuable and far better compared with what was previously planned and strongly recommended the program continue.

### Participating in Resident Scholarly Activity

Over the course of the pilot, the participating resident noted involvement in 3 active research projects that aligned with the focused training experience in aesthetic surgery. Two manuscripts/chapters were published in peer-reviewed journals, and 1 oral presentation was given at a national meeting during that timeframe. The participating resident scored in the first quartile on the breast and cosmetic section of the ISE as a PGY5; however, as a PGY6, following the focused training experience, this resident scored in the fourth quartile on the same section—representing a substantial improvement.

### Participating Resident Case Logs

Because of the nature of our program, the experience was predominantly centered around aesthetic surgery of the head and neck region. The complete case logs for the pilot are included (Appendix D). During the 6-month pilot, the participating aesthetic resident completed 204 cases overall, including procedures performed while fulfilling call and other service obligations. In terms of the focused training experience, this included cases in facial aesthetics as well as aesthetic procedures of the breast and body (Table 2).

**Table 2.** Participating Resident Case Log (Aesthetic Cases)

Facial aesthetics	125
Face and neck lift	21
Brow lift	9
Blepharoplasty (upper and/or lower)	26
Rhinoplasty	27
Other “aesthetic deformities of the head and neck”^a^	42
Breast and body	13
Mastopexy	2
Brachioplasty	6
Abdominoplasty	2
Suction assisted lipectomy	3

^a^Categorization denoted and assigned within the Accreditation Council for Graduate Medical Education Case Log System.

## DISCUSSION

The plastic surgery resident selection process prioritizes academic excellence. This includes but is not limited to (1) outstanding performance on the United States Medical Licensing Examination Step 2, (2) publications in peer-reviewed journals, (3) Alpha Omega Alpha status, and (4) excellent letters of recommendation. A dedicated research year before applying has become commonplace.^3^ This clearly results in an excellent prospective resident application pool.

Resident training itself emphasizes exposure and proficiency in a broad array of complex areas of plastic surgery. The current integrated plastic surgery model includes 6 years of training in the various general plastic and subspecialty surgical areas. Currently, ∼50% of integrated residents then choose 1 year of additional optional subspecialty fellowship training.^4,5^ Because plastic surgery is so diverse, many plastic surgeons ultimately focus their careers in a given subspecialty area.

Several recent innovative pilots have been initiated with approval from the ABPS. CBE is an effort to dictate training not by program length but by resident skillset and knowledge base. Although not designed specifically to reduce training time, this offers the resident the possibility of either entering practice 1 year early or completing both plastic surgery residency and postgraduate subspecialty training within a 6-year time frame.^1^ Thus, the graduate may obtain ABPS eligibility as well as an additional subspecialty fellowship year if desired within the current 6-year integrated training period.

However, CBE does have its drawbacks. These include the need for new teaching strategies, additional administrative burdens, scheduling logistics, difficulty correlating the 5 years of training with fellowship applications, and cost.^1^ Finally, the program pilot is most appropriate for a limited number of larger programs where loss of a resident position would be less disruptive than it would be to a smaller program. Our pilot avoided such potential disruptions in education and service because the focused training resident remained fully involved in all conferences and call schedules. That the focused training did not disrupt resident education was confirmed by positive responses of 95% of our nonparticipating residents.

Most recently, orthopedic and plastic surgery have formalized the concept of an embedded hand fellowship, allowing residents in participating programs to spend their entire sixth year in hand training at their home institution. Graduating residents would then be able to sit for both ABPS examinations and for a certificate of added qualification in hand surgery. This is currently being piloted at programs such as Washington University in St Louis and Southern Illinois University. Although this hand fellowship pilot represents a significant advancement in the education of the hand surgery trainee, it addresses only one of the many subspecialty opportunities open to plastic surgery residents. Our pilot offers the possibility of opening advanced training in a number of other subspecialty areas. The factor limiting resident choice of focused training, of course, will vary from program to program and may be limited by the depth and diversity of cases available. Specifically, in our abbreviated pilot, our senior resident was torn between 2 potential fellowships: craniofacial and aesthetic surgery. Focused training was an ideal solution in this case. He spent 6 months in focused training in aesthetic surgery and then went on to do a yearlong craniofacial surgery fellowship. This resident dilemma of the need to choose 1 subspecialty over another may not be unique. When surveyed, nearly 50% of our yet-to-participate residents stated that they would choose an area of focused training different from their planned postgraduate fellowship.

A review of the literature for additional articles pertaining to focused training or embedded fellowships during one's residency yields little additional information. Neurosurgery is the exception. Neurosurgical residency training has successfully implemented enfolded fellowships in subspecialty areas such as epilepsy surgery, neurocritical care, and endovascular neurosurgery, among others.^2^

A number of the plastic surgery subspecialties have recently broadened their scope of practice. A number of craniofacial fellowships now include facial feminization and complex orthognathic surgery as an integral part of their training, whereas microsurgery has incorporated lymphedema treatment and super-microsurgery into its armamentarium.^6^ Aesthetic surgery has expanded its reach with the addition of minimally invasive techniques and aesthetic medicine.^7^ Focused training at certain institutions may offer residents early exposure to these newer areas and better prepare them for either their upcoming fellowship or their future practice.

Numerous studies investigating the perception of aesthetic training in residency have suggested that although plastic surgery residents are quite confident in breast and body procedures, there is a desire for additional residency training in the facial aesthetic areas, including rhinoplasty, facelift, and minimally invasive techniques. In the case of our first participating resident, this was addressed through our focused training effort and corroborated by our posttraining survey.^7-9^

Objective measures documenting the efficacy or success of a given program are both essential and exceedingly difficult. We have attempted to provide objective evaluation measures to gauge potential progress in the program, including anonymous staff and resident evaluations, focused plastic surgery operative logs, and research performance analysis. Although some may call this objectifying the subjective, this is quite similar to evaluations of other plastic surgery initiatives. The limitations of our participating resident survey should be noted because of its lack of anonymity.

Studies that seek to examine predictors for success after graduating plastic surgery residency are unfortunately lacking. This may very well be a function of the fact that definitions of success vary from person to person and the challenges associated with attempting such research. Although an in-depth discussion of the best means to objectively measure long-term success following residency is beyond the scope of this paper, it is suggested that 1 critical component will be the strength and quality of one's plastic surgery training. The contemporary landscape of plastic surgery practice has changed substantially in recent years and will continue to do so. Competition has increased, and a number of specialties have encroached on the territory of organized plastic surgery. In the business world, failure to evolve risks financial and reputation loss at best and obsolescence at worst.^10^ We offer the concept of focused training as both a possible means of enhancing plastic surgery training and better preparing our graduates for future success. Although our pilot is in evolution and success is far from assured, it should be noted that both participating and nonparticipating residents were enthusiastic regarding the pilot. When surveyed, all junior residents noted that they were anxious to participate, and all residents agreed that the pilot should continue.

One final consideration pertains to how the focused training initiative may facilitate recruitment of prospective applicants. Previous research has suggested that recent ratios of applicants to available positions pose concerns and that cultivating more opportunities to attract medical students to plastic surgery residency remains a desirable goal.^11^ Others have indicated that when selecting a program, medical students value interview day impressions, their experience during away rotations, support and mentoring on behalf of the faculty, personal interactions with the residents, as well as the amount of time spent on general surgery services.^12^ At our institution, during this year's residency interviews, we noticed that nearly two-thirds of all medical students asked about and expressed interest in the focused training experience. It may very well be that this initiative has increased desirability from the perspective of the applicant; however, more research is needed before arriving at a definitive conclusion.

Our objective assessment demonstrates that adjusting the traditional residency paradigm during the final year to allow for a focused training experience was favorably received by all residents and participating staff. Attending plastic surgeons unanimously agreed that the pilot offered a substantial advantage and improvement to the current residency training model and supported its continuation. Notably, there were no major criticisms, problems, or administrative burdens encountered. Some may be concerned that 6 months may be too long for a focused experience; however, this represents an arbitrary number, and if other programs feel that a shorter time is more appropriate, it could certainly be tailored.

Key limitations include the need to develop modules beyond aesthetics as well as the fact that this initiative may not be ideal for every program. Although only a single resident completed the program, it is possible that a different individual would have had different suitability. It is not only the single response to the survey that is impacted, but perhaps this was just 1 particular resident who made the program successful. We must also emphasize that initiating the pilot with an aesthetics module was not intended to disregard the desires of others or to bypass programs with a limited ability to participate in a similar fashion. The aesthetic module was simply the most straightforward way to attempt implementation and objectification of this concept at our institution. We also acknowledge that focused training in any subspecialty area requires significant volume and case diversity to be a viable option.

The diversity of possible areas of focused training will be limited by the particular strengths of individual programs, with different programs able to have different focused experiences across the various subspecialties. In addition, noted challenges exist when multiple residents wish to select the same area of focus. One possible weakness is that programs offering focused training may become more desirable, and those that do not, may be less so. This represents a potential political risk to smaller programs and could create a multitiered system. Finally, the value of the participating resident survey might be questioned. Because only a single resident participated in the survey, it could not be considered a blinded response. Postgraduate follow-up with the participating resident (who ultimately completed a craniomaxillofacial fellowship) revealed that the focused experience was still felt to represent a valuable asset to subsequent postgraduate training and early practice. Perhaps a future consideration might be to allow residents to rotate at other plastic surgery programs for their focused training experience. However, this may be logistically difficult or unfeasible given specific institutional resident rules, malpractice issues, and service limitations.

## CONCLUSIONS

The concept of focused training in plastic surgery is proposed as a pilot project. It is designed to allow a given resident the opportunity to spend 6 months of the sixth training year in an area of his/her specific subspecialty interest. Results of our limited pilot demonstrated overwhelming enthusiasm for the concept, as noted by anonymous surveys from participating residents, nonparticipating residents, and staff. Further, anonymous surveys of nonparticipating residents found no adverse effect on their resident experience, including caseload or service issues. Potential benefits include the ability to permit residents to focus on an area of their passion, the avoidance of “senioritis” (loss of enthusiasm and motivation for the day-to-day activities of residency), an enhanced level of sophistication and preparation for plastic surgery practice, increased resident academic productivity, and a significant interest to medical student applicants. Ultimately, the need to develop additional modules remains necessary.

## Supplementary Material

ojaf040_Supplementary_Data
