# Supplementary material for: Focused Subspecialty Training in Plastic Surgery Residency: An Objective Assessment of the Cleveland Clinic Pilot Program
Source: Aesthet Surg J Open Forum. 2025 May 12;7:ojaf040. doi: 10.1093/asjof/ojaf040 (PMC12202876; doi:10.1093/asjof/ojaf040)
Supplement: ojaf040_Supplementary_Data [file ojaf040_supplementary_data.zip › revSDC2.pdf]

Cleveland Clinic Focused Training in Plastic Surgery Pilot Program: Resident Survey

21	03:33	Active
Responses	Average time to complete	Status

1. What is your current PGY year?

2	5
3	4
4	4
5	4
6	4

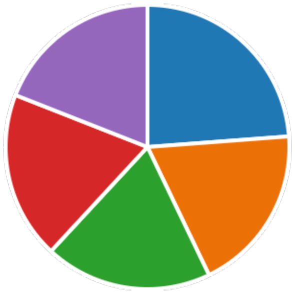

2. Are you planning to pursue **formal** post-residency fellowship training?

Yes	15
No	6

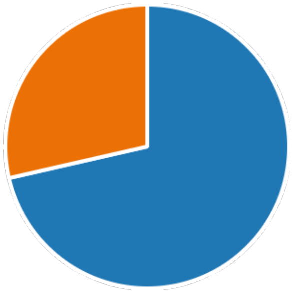

3. If you answered YES to the previous question, please specify the specialty of interest you are most likely to pursue

Aesthetics	5
Craniofacial	3
Hand	3
Microsurgery	2
Transgender	1
Other	1

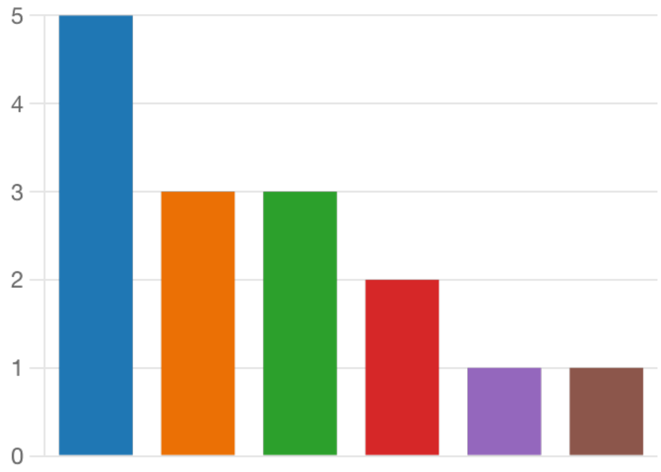

4. Would you be interested in participating in a Focused Subspecialty Training opportunity during your chief year?

Yes	21
No	0

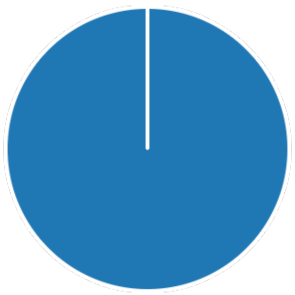

5. If you answered YES to the previous question, please specify the specialty of interest you are most likely to pursue

Aesthetics	14
Craniofacial	1
Hand	2
Microsurgery	0
Transgender	1
Other	3

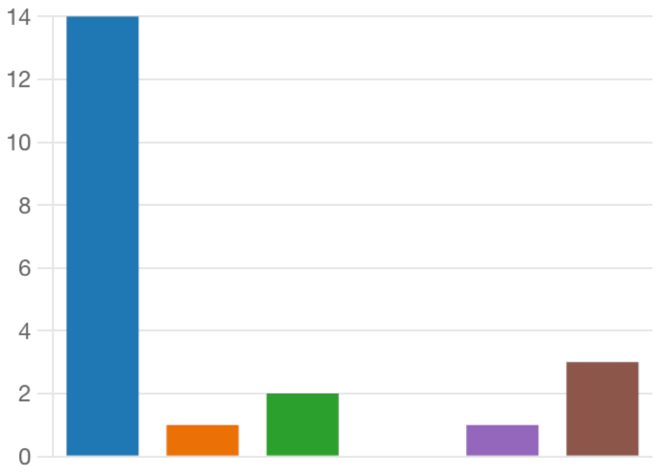

6. Would you be interested in completing a formal post-graduate fellowship in a **different** area than your anticipated Focused Subspecialty Training opportunity?

Yes	10
No	11

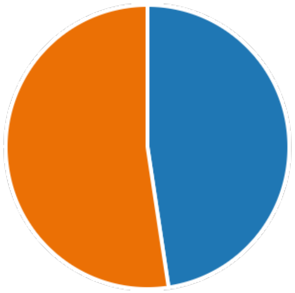

7. If you answered YES to the previous question, please specify the specialty of interest you are most likely to pursue

Aesthetics	4
Craniofacial	2
Hand	1
Microsurgery	1
Transgender	0
Other	2

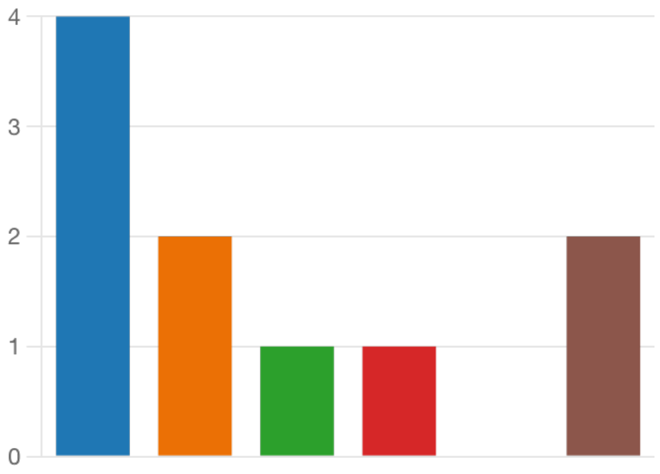

8. Would you be interested in completing a formal post-graduate fellowship in a **same** area than your anticipated Focused Subspecialty Training opportunity?

Yes	8
No	13

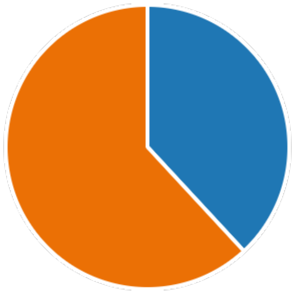

9. If you answered YES to the previous question, please specify the specialty of interest you are most likely to pursue

Aesthetics	5
Craniofacial	0
Hand	2
Microsurgery	0
Transgender	1
Other	0

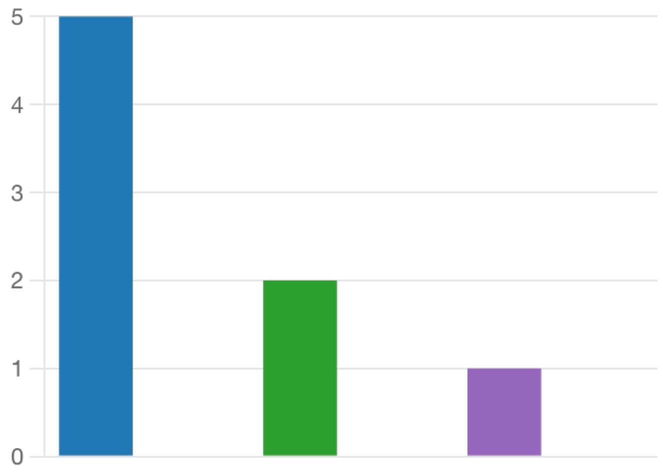

10. This year's Focused Training Pilot Program created additional work and/or stress related to my daily routine

Never	17
Rarely	3
Sometimes	1
Often	0
Nearly always	0

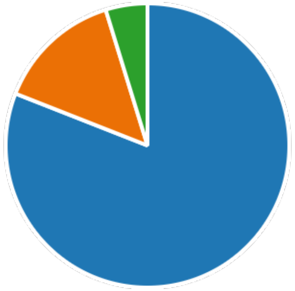

11. Would you recommend the Focused Training Program continue?

Yes	21
No	0

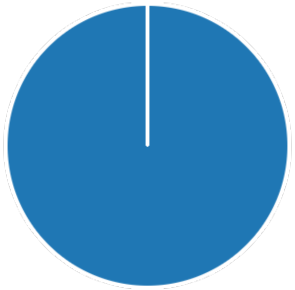

12. What is your impression of the focused training model compared to the traditional 6th year of residency training?

Neutral	1
Somewhat better	2
Better	8
Far better	10

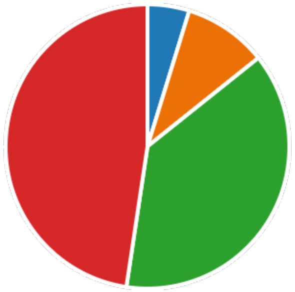

13. Please enter any comments you have, including those related to the questions above. Thank you for participating.

Please refer to Table 1 for a complete listing of responses.
