# Supplementary material for: Focused Subspecialty Training in Plastic Surgery Residency: An Objective Assessment of the Cleveland Clinic Pilot Program
Source: Aesthet Surg J Open Forum. 2025 May 12;7:ojaf040. doi: 10.1093/asjof/ojaf040 (PMC12202876; doi:10.1093/asjof/ojaf040)
Supplement: ojaf040_Supplementary_Data [file ojaf040_supplementary_data.zip › revSDC3.pdf]

Cleveland Clinic Focused Training in Plastic Surgery Pilot Program: Staff Survey

Responses	Average time to complete	Status
6	07:50	Active

1. The Focused Subspecialty Training pilot enhanced the PGY6 resident's experience

Strongly disagree	0
Disagree	0
Agree	4
Strongly agree	2

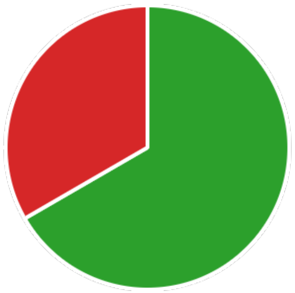

2. The Focused Subspecialty Training pilot offers a significant advance/improvement compared to the current integrated residency training program

Strongly disagree	0
Disagree	0
Agree	4
Strongly agree	2

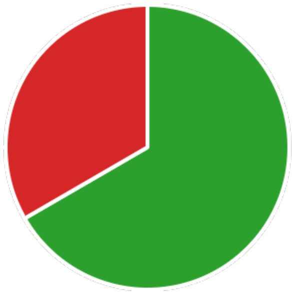

3. Would you recommend the program continue?

Yes	6
No	0

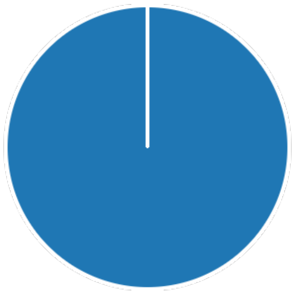

4. What is your overall assessment of the pilot program?

Please refer to Table 1 for a complete listing of responses.

5. What suggestions do you have for improvement?

Please refer to Table 1 for a complete listing of responses.
