# Supplementary material for: Focused Subspecialty Training in Plastic Surgery Residency: An Objective Assessment of the Cleveland Clinic Pilot Program
Source: Aesthet Surg J Open Forum. 2025 May 12;7:ojaf040. doi: 10.1093/asjof/ojaf040 (PMC12202876; doi:10.1093/asjof/ojaf040)
Supplement: ojaf040_Supplementary_Data [file ojaf040_supplementary_data.zip › SDC1.pdf]

Focused Subspecialty Training Program in Aesthetic Surgery
Cleveland Clinic Pilot

Project Description:

Aesthetic surgery plays a vital role in many ABPS diplomats' clinical practice. Recent estimates suggest that 50% of board-certified plastic surgeons incorporate some aspect of cosmetic surgery into their practice. At the same time competition from outside plastic surgery has increased significantly as has a level of sophisticated surgical techniques. Minimally invasive surgery and cosmetic medicine have also made significant inroads in the field.

If Plastic Surgery is to maintain its leadership role plastic surgeons and trainees will need to keep abreast of ongoing advances. Plastic surgery resident education requires exposure to a broad range of surgical disciplines and increasingly complex surgical problems. Cosmetic surgery is just one of many areas to master and time given to one means time taken away from another. Facial cosmetic surgery, in particular, has been noted by some to be an area of training weakness.

One means of increasing the level of sophisticated knowledge base and technical expertise in facial aesthetics would be to provide a focused and in-depth experience to residents interested in the field. A focused subspecialty training opportunity in the sixth year of integrated plastic surgery resident education is therefore proposed.

Description of Innovation:

For residents to qualify for the Focused Subspecialty Training opportunity they must meet the following criteria:

1. Successful completion of all minimum numbers of index cases in the Plastic Surgery Operative Log (PSOL) by the end of their fifth year.
2. Satisfactory progression through ACGME milestones and demonstration of satisfactory graded responsibility as assessed by the Program Director (to at least Level 4).
3. Documentation of quality interpersonal skills as evidenced by peers, nursing, and ancillary personnel through the 360° evaluation process.
4. Acceptable performance on the Annual In-service Examination as deemed by the Program Director (e.g., 50th percentile averaged over the previous 5 years)

With the above criteria met it is proposed that the resident may enter the embedded six-month focused subspecialty training opportunity to increase the level of sophistication in all aspects of aesthetic medicine and surgery.

A standardized didactic curriculum has been established (Addendum 1).

Focused Subspecialty Training structure would include the following:

1. Four days of surgery and clinic time per block diagram.
2. One day of protected clinical research time.

3. Bi-monthly presentation of advanced aesthetic didactic conference presented to residents and staff.
4. Active participation in bi-weekly aesthetic research conference
5. Submission of a minimum of one abstract to ASPS, ASAPS, or AAPS during the six-month period.
6. Organization of a quarterly virtual journal club open to residents, fellows and students nationally.
7. Submission of a completed Plastic Surgery Operative Log (PSOL) at the end of the experience.

Goals and Objectives:

1. To increase resident level of sophistication and knowledge base in the area of facial aesthetic surgery. This is to include, but is not limited to preoperative evaluation, surgical and nonsurgical alternatives in the treatment of complications.
2. To assure the patient received the highest level of quality care with minimal morbidity.
3. To assure that plastic surgery training is of the highest possible quality.
4. To actively and objectively measure progressive resident learning through use of interactive case management between staff and resident, ACGME milestones, and 360° evaluations.

Anticipated Outcomes:

Given the opportunity to focus exclusively on aesthetic medicine and surgery it is expected that at the completion of the experience the resident will have a significant increase in knowledge and a more nuanced approach to the aesthetic patient. This ultimately should lead to enhanced patient care.

Methodology and Evaluation:

The proposed pilot is expected to improve the learning environment and resident education not only for the resident in the experience but also for residents in general. As the participating resident increases his/her level of sophistication this will be shared with other residents through the advanced lecture series, the interaction during standard resident cosmetic lectures, and through optional teaching endeavors, such as cadaver dissections and resident clinics

Curriculum Variables:

Should a number of residents apply, not all will be able to participate. A maximum of two residents will be able to participate per year. Therefore the program director may be in a position to have to select one resident over another.

Since the focused subspecialty training opportunity will be located at the home institution, the participating resident will also fully participate in call responsibilities.

Assessment of Program:
Assessment tools for resident evaluation will include those used throughout the residency and include assessment of the five competencies and evaluation by the CCC. Additional assessment tools specific to the experience will include director/faculty evaluation. Heightened performance regarding patient evaluation and technical abilities will be expected to approach fellowship level rather than senior resident level of performance.

A table below addresses this assessment and methods of targeted competencies:

Assessment Tool	Targeted Competency
Faculty Evaluation	Multiple Competency
Direct Observation	Patient Care, Communication, Medical Knowledge
Multi-Service Feedback	Practice Based Learning, System Based Practice
Simulation	Procedures

How will the Pilot Enhance Patient Outcomes?
A focused six-month experience is expected to result in a resident product that has a more nuanced and sophisticated approach to problems in aesthetic surgery, better answers to patient issues, and greater choices for patient's aesthetic concerns.

How Will the Pilot Enhance Learner Outcomes?
The focused experience will allow greater attention to cosmetic details, greater opportunity to increase aesthetic fund of knowledge and provide the opportunity for development of a more nuanced and effective product.

Describe faculty development approaches that will ensure participating faculty members understand the goals and objectives of the pilot and can effectively use the pilot assessment tools/methods required for affective programmatic assessment:

Sharing goals and objectives with participating faculty will be the responsibility of the focused subspecialty director. This will include an initial meeting prior to the commencement of the experience and required attendance at advance didactic lectures led by the participating resident. Tools utilized to assess resident progress will be the same as those used for other residents. However, a heightened level of understanding and performance approaching that of the fellowship level is to be expected as the resident progresses through the focused training program. Scientific rigor will be expected of the aforementioned research project(s).

Monitoring:

Program evaluation will be accomplished using blinded resident and faculty surveys midway through and at the completion of the experience. Both participating and non-participating faculty will be queried.

Timeline:
If approved, the pilot will begin July 1, 2022. If evaluations prove positive, the pilot could become part of the expected curriculum.

Criteria for Assessing Success:
Positive survey results from participating and non-participating residents and faculty would represent high indicators of success. Improvement in the aesthetic section of the annual In-Service Examination for those beginning their focused experience in July would be seen as a positive development. Finally an increase in scholarly activity would also be seen as a high indicator.

Conclusion:
Aesthetic surgery is an important component of a plastic surgeon's practice. There is a consensus among Directors of the American Board of Plastic Surgery (ABPS) that training in aesthetic surgery could be improved. An experience in facial aesthetic surgery at the senior resident level for those who are interested is seen as a means of improving this deficiency. A pilot project which includes a six-month focused subspecialty training experience in aesthetic surgery is therefore proposed.

Curriculum for the Focused Subspecialty Training Experience in Facial Aesthetic Surgery (Addendum 1)

1. Aesthetic Principles of the Face
    - A. Forehead and Temporal Region
    - B. Mid Face Muscles, Ligaments, Facial Nerve
    - C. Lower Face
    - D. Fat Compartments Superficial and Deep
    - E. Superficial, Intermediate and Deep Neck Structures

2. Upper Face
    - A. Brow/Lower Lid Proportions and Analysis
    - B. Brow Lifting Techniques
        1. Noninvasive
        2. Endoscopic
        3. Direct
        4. Hairline
        5. Temporal

3. Upper Eyelid
    A. Analysis
    B. Eyelid Ptosis
    C. Technique

4. Lower Eyelid
    A. Lower Eyelid Analysis and Pre-op Evaluation
    B. Lower Eyelid Techniques
        1. Skin only
        2. Transconjunctival
        3. Orbicularis Repositioning
        4. Canthal Tightening

5. Mid Face
    A. Open
    B. Closed
    C. Non-invasive Volumetric Augmentation

6. Face and Neck
    A. Analysis
    B. Pre-operative Planning
    C. Operative Techniques
        1. Anterior only approach
        2. Platysmaplasty
        3. Facelift
            A. SMAS plication
            B. SMASectomy
            C. Extended SMAS
            D. Composite

7. Rhinoplasty
    A. Analysis
    B. Structural Considerations
    C. Techniques
        1. Open vs. Closed
        2. Incisions
        3. Grafts
        4. Primary Rhinoplasty
        5. Secondary Rhinoplasty
        6. Airway Obstruction
            A. Surgical Management
            B. Non-Surgical Management

8. Alopecia/Hair Transplantation/Restoration
    A. Principles
    B. Surgical Techniques

C. Clinical Applications
D. Complications and Management

9. Cosmetic Medicine
   A. Soft Tissue Augmentation
      1. Synthetic Fillers
      2. Fat Transfer
         A. Physiology and Techniques
      3. Resurfacing
         A. Biophysics
         B. Lasers
         C. Chemical Peels
         D. Complications
      4. Skin Care (including PRS operative preparation for laser, chemical peels)

10. The Practice of Aesthetic Surgery
   A. Outpatient Office Management
   B. Medical Photography
   C. Medical Legal Aspects
