# Supplementary material for: Focused Subspecialty Training in Plastic Surgery Residency: An Objective Assessment of the Cleveland Clinic Pilot Program
Source: Aesthet Surg J Open Forum. 2025 May 12;7:ojaf040. doi: 10.1093/asjof/ojaf040 (PMC12202876; doi:10.1093/asjof/ojaf040)
Supplement: ojaf040_Supplementary_Data [file ojaf040_supplementary_data.zip › SDC4.pdf]

ACGME

Cleveland Clinic Foundation Program
Resident
For Surgeon / All Patient Types / All Rotations
Done between 7/1/2022 and 12/31/2022

	Year 1	Year 2	Year 3	Year 4	Year 5	Year 6	Total
**HEAD AND NECK CONGENITAL DEFECTS**							
Primary cleft lip repair	0	0	0	0	0	0	0
Primary cleft palate repair	0	0	0	0	0	0	0
Secondary cleft lip or palate repair	0	0	0	0	0	1	1
Cleft lip nasal deformity repair	0	0	0	0	0	1	1
Craniomaxillofacial reconstruction	0	0	0	0	0	0	0
Vascular malformation (laser)	0	0	0	0	0	0	0
Other (Head and Neck Congenital Defects)	0	0	0	0	0	1	1
**Total HEAD AND NECK CONGENITAL DEFECTS**	0	0	0	0	0	3	3
**HEAD AND NECK NEOPLASMS**							
Reconstruction of defect after neoplasm resection with skin graft	0	0	0	0	0	1	1
Reconstruction of defect after neoplasm resection with local flap	0	0	0	0	0	1	1
Reconstruction of defect after neoplasm resection with free flap	0	0	0	0	0	0	0
Resection of skin cancer	0	0	0	0	0	2	2
Resection of other head and neck neoplasm	0	0	0	0	0	0	0
Other (Head and Neck Neoplasms)	0	0	0	0	0	4	4
**Total HEAD AND NECK NEOPLASMS**	0	0	0	0	0	8	8
**HEAD AND NECK TRAUMA**							
Treat occlusal injury	0	0	0	0	0	0	0
Treat upper midface fracture	0	0	0	0	0	0	0
Treat nasal fracture	0	0	0	0	0	0	0
Treat complex soft tissue injury	0	0	0	0	0	3	3
Other (Head and Neck Trauma)	0	0	0	0	0	10	10
**Total HEAD AND NECK TRAUMA**	0	0	0	0	0	13	13
**BREAST MACROMASTIA**							
Breast Reduction	0	0	0	0	0	1	1
**Total BREAST MACROMASTIA**	0	0	0	0	0	1	1
**ABSENT BREAST**							
Breast reconstruction with implant	0	0	0	0	0	0	0
Breast reconstruction with pedicle flap	0	0	0	0	0	0	0
Breast reconstruction with free flap	0	0	0	0	0	0	0

Fat grafting (absent breast)	0	0	0	0	0	0	0
**Total ABSENT BREAST**	0	0	0	0	0	0	0
**OTHER DEFORMITIES OF BREAST**							
Treat other deformities	0	0	0	0	0	0	0
**Total OTHER DEFORMITIES OF BREAST**	0	0	0	0	0	0	0
**WOUNDS OR DEFORMITIES OF TRUNK**							
Treat pressure ulcer	0	0	0	0	0	0	0
Treat pressure ulcer with flap	0	0	0	0	0	0	0
Treat wounds of trunk with flap	0	0	0	0	0	2	2
**Total WOUNDS OR DEFORMITIES OF TRUNK**	0	0	0	0	0	2	2
**OTHER DEFORMITIES OR DISEASE PROCESSES OF TRUNK**							
Treat other deformities	0	0	0	0	0	3	3
**Total OTHER DEFORMITIES OR DISEASE PROCESSES OF TRUNK**	0	0	0	0	0	3	3
**HAND AND UPPER EXTREMITY REQUIRING RECON**							
Reconstruction by primary closure	0	0	0	0	0	0	0
Reconstruction with skin graft	0	0	0	0	0	0	0
Reconstruction with flap	0	0	0	0	0	0	0
Amputation	0	0	0	0	0	0	0
**Total HAND AND UPPER EXTREMITY REQUIRING RECON**	0	0	0	0	0	0	0
**TENDON (EXTENSOR OR FLEXOR)**							
Repair/reconstruct tendon with or without graft	0	0	0	0	0	0	0
Operative release of tendon adhesion/tendon lengthening	0	0	0	0	0	0	0
Tendon Transfer	0	0	0	0	0	0	0
**Total TENDON (EXTENSOR OR FLEXOR)**	0	0	0	0	0	0	0
**NERVE INJURY**							
Repair/reconstruct nerve with or without graft	0	0	0	0	0	0	0
**Total NERVE INJURY**	0	0	0	0	0	0	0
**FRACTURE OR DISLOCATION**							
Operative repair of fracture or dislocation	0	0	0	0	0	4	4
Release of joint contracture	0	0	0	0	0	0	0
**Total FRACTURE OR DISLOCATION**	0	0	0	0	0	4	4

**DUPUYTREN'S CONTRACTURE**							
Operative treatment of Dupuytren’s contracture	0	0	0	0	0	0	0
**Total DUPUYTREN'S CONTRACTURE**	0	0	0	0	0	0	0
**NERVE COMPRESSION**							
Nerve decompression	0	0	0	0	0	3	3
**Total NERVE COMPRESSION**	0	0	0	0	0	3	3
**ARTERIAL INSUFFICIENCY OR AMPUTATION–UPPER EXTREM**							
Arterial repair, revascularization, or replantation of digit, hand, or upper extremity	0	0	0	0	0	0	0
**Total ARTERIAL INSUFFICIENCY OR AMPUTATION–UPPER EXTREM**	0	0	0	0	0	0	0
**OTHER DEFORMITY OR DISEASE PROCESS**							
Arthroplasty/arthrodesis	0	0	0	0	0	0	0
Treat congenital deformity	0	0	0	0	0	0	0
Treat neoplasm (benign or malignant)	0	0	0	0	0	0	0
Other (Other Deformity or Disease Process)	0	0	0	0	0	2	2
**Total OTHER DEFORMITY OR DISEASE PROCESS**	0	0	0	0	0	2	2
**LOWER EXTREMITY WOUNDS AND DEFORMITIES**							
Treatment with graft	0	0	0	0	0	0	0
Treatment with local flap	0	0	0	0	0	0	0
Treatment with free flap or revascularization/replantation	0	0	0	0	0	0	0
**Total LOWER EXTREMITY WOUNDS AND DEFORMITIES**	0	0	0	0	0	0	0
**OTHER DEFORMITIES OF LOWER EXTREMITY**							
Treat other deformities	0	0	0	0	0	0	0
**Total OTHER DEFORMITIES OF LOWER EXTREMITY**	0	0	0	0	0	0	0
**INTEGUMENT BURNS**							
Burn reconstruction	0	0	0	0	0	0	0
Other (Integument Burns)	0	0	0	0	0	0	0
**Total INTEGUMENT BURNS**	0	0	0	0	0	0	0
**LESIONS OF THE INTEGUMENT**							
Treat benign lesions	0	0	0	0	0	1	1
Treat malignant lesions	0	0	0	0	0	0	0

INTEGUMENT

**WOUNDS AND OTHER LESIONS OF THE INTEGUMENT**							
Treat deformities	0	0	0	0	0	0	0
**Total WOUNDS AND OTHER LESIONS OF THE INTEGUMENT**	0	0	0	0	0	0	0
**HEAD AND NECK AESTHETIC DEFORMITY**							
Facelift	0	0	0	0	0	21	21
Browlift	0	0	0	0	0	9	9
Blepharoplasty	0	0	0	0	0	26	26
Rhinoplasty	0	0	0	0	0	27	27
Other (H&N Aesthetic Deformity)	0	0	0	0	0	42	42
**Total HEAD AND NECK AESTHETIC DEFORMITY**	0	0	0	0	0	125	125
**BREAST MICROMASTIA**							
Breast augmentation	0	0	0	0	0	0	0
Fat grafting (breast micromastia)	0	0	0	0	0	0	0
**Total BREAST MICROMASTIA**	0	0	0	0	0	0	0
**BREAST PTOSIS**							
Mastopexy	0	0	0	0	0	2	2
**Total BREAST PTOSIS**	0	0	0	0	0	2	2
**OTHER DEFORMITIES OF BREAST**							
Other (Other Deformities of Breast)	0	0	0	0	0	0	0
**Total OTHER DEFORMITIES OF BREAST**	0	0	0	0	0	0	0
**TRUNK/EXTREMITY AESTHETIC DEFORMITIES**							
Brachioplasty	0	0	0	0	0	6	6
Abdominoplasty	0	0	0	0	0	2	2
Body lift	0	0	0	0	0	0	0
Thighplasty	0	0	0	0	0	0	0
Suction assisted lipoplasty	0	0	0	0	0	3	3
Other (Trunk/Extremity Aesthetic Deformities)	0	0	0	0	0	1	1
**Total TRUNK/EXTREMITY AESTHETIC DEFORMITIES**	0	0	0	0	0	12	12
**ADDITIONAL PROCEDURES (NON-INDEX)**							
Botulinum toxin injection	0	0	0	0	0	5	5

**Total ADDITIONAL PROCEDURES (NON-INDEX)**	0	0	0	0	0	14	14
**Core Surgery**							
Alimentary Tract/Abdominal Surgery	0	0	0	0	0	0	0
Breast Surgery/Other Oncologic Tumor Surgery	0	0	0	0	0	0	0
Trauma/Critical Care/Anesthesia Procedures	0	0	0	0	0	0	0
Other Surgery	0	0	0	0	0	7	7
**Total Core Surgery**	0	0	0	0	0	7	7
**Non-Tracked Codes**							
Non-Tracked Codes	0	0	0	0	0	4	4
**Total Non-Tracked Codes**	0	0	0	0	0	4	4
**GRAND TOTAL**	0	0	0	0	0	204	204
